# Recent initiatives on guidelines implementation: KT in primary care

**DOI:** 10.1186/1710-1492-6-S4-A12

**Published:** 2010-12-10

**Authors:** Louis-Philippe Boulet

**Affiliations:** 1 Institut universitaire de cardiologie et de pneumologie de Québec, Laval University Chair on Knowledge Transfer, Education and Prevention in Respiratory and Cardiovascular Health, Québec, Québec, G1V 4G5, Canada

## 

In Canada, numerous initiatives have been developed in the recent years to improve guidelines implementation in respiratory care. Following the Canadian Thoracic Society (CTS) *1999 Canadian Asthma Consensus Guideline* (CACG), efforts have been devoted to better disseminate this document and foster its implementation. This included among others, the production of a series of publications, development of a web-site, mailings of key messages to primary care physicians, interactive workshops and integration of CACG recommendations in educational programs.

In the past, Canadian respiratory health guidelines have been produced and disseminated according to various agendas and methods, but in the last few years, the CTS has decided to develop a common body, the *Canadian Respiratory Guidelines Committee* (CRGC). The goal of the CRGC is to produce, disseminate, help implement and evaluate Canadian respiratory guidelines according to uniform methods and in a collaborative fashion, with a common annual agenda [1]. The methods used to produce and assess the performance of those guidelines are described in the publication by Gupta *et al*. [2]. Hopefully, this new strategy will allow a better use of resources and a more efficient translation process. Initiatives aimed at improving guideline implementation have also been developed and a specific *Dissemination and Implementation Subcommittee* has been formed to address this. A large scale project, the “Guidelines Implementation in Primary Care” (GIPC) study, based on *quality circles* and mentorship for primary care physicians (PCP) and involving interactive sessions and the use of *practice tools* had been developed but faced difficulties in regard to physicians recruitment, stressing the challenges associated in the involvement of busy PCPs is such studies. Various other means of contributing to guidelines implementation are presently considered. Documents and tools to facilitate the translation of guidelines can be found at www.respiratoryguidelines.ca.

There is, therefore, a need to promote implementation initiatives according to the most effective methods recognized and to develop innovative strategies to improve the translation of guidelines recommendations, while considering the cost-effectiveness of these interventions in addition to the needs and motivations of the targeted groups. This is also one of the key mandates of the recently developed Laval University Chair on Knowledge Transfer, Education and Prevention in Respiratory and Cardiovascular Health, and various means of translating current cardio-respiratory guidelines are being developed and will soon be available on the site www.coeurpoumons.ca.

Finally, numerous other asthma guidelines implementation initiatives have been proposed in many Canadian provinces in the last two decades (the reader is invited to consult their web site at http://www.lung.ca/about-propos/provincial-provinciales_e.php), and additional efforts are currently devoted to improving the effectiveness of such interventions. More recently, the National Lung Health Framework [3] has been developed, and should help address many of the remaining care gaps and target populations most in need of such interventions. The “Framework” is an action plan developed by and for a wide range of stakeholders working to improve lung health in Canada, using a collaborative approach to the prevention and management of respiratory disease. Finally, many educational and support networks such as the Canadian Network for Respiratory Care (http://www.cnrchome.net/), the Quebec Asthma and COPD Network (http://rqam.ca/), patient support groups and various other organizations, are contributing to the respiratory guidelines implementation through their educational programs.

Hopefully, learning from the past, inspired by other countries’ initiatives and acting in collaboration with all stakeholders, we will be able to build and maintain a successful long-term guidelines implementation program that contributes to the improvement of respiratory health in Canada.
